# Hospital costs of robotic-assisted and open treatment of large ventral hernias

**DOI:** 10.1038/s41598-024-62550-w

**Published:** 2024-05-21

**Authors:** Flaminia Sabbatini, Davide La Regina, Nicole Murgante Testa, Anna Maria Senatore, Andrea Saporito, Ramon Pini, Francesco Mongelli

**Affiliations:** 1 Department of Surgery, Bellinzona e Valli Regional Hospital, EOC, Via Gallino 12, 6500 Bellinzona, Switzerland; 2 Faculty of Biomedical Sciences, Università Della Svizzera Italiana, Via La Santa 1, 6900 Lugano, Switzerland; 3 Department of Anesthesia, Bellinzona e Valli Regional Hospital, EOC, Via Gallino 12, 6500 Bellinzona, Switzerland

**Keywords:** Incisional hernia, Ventral hernia, Robotic, Surgery, Costs, Intestinal diseases, Gastroenterology, Health care

## Abstract

Robotic-assisted treatment of ventral hernia offers many advantages, however, studies reported higher costs for robotic surgery compared to other surgical techniques. We aimed at comparing hospital costs in patients undergoing large ventral hernia repair with either robotic or open surgery. We searched from a prospectively maintained database patients who underwent robotic or open surgery for the treatment of the large ventral hernias from January 2016 to December 2022. The primary endpoint was to assess costs in both groups. For eligible patients, data was extracted and analyzed using a propensity score-matching. Sixty-seven patients were retrieved from our database. Thirty-four underwent robotic-assisted surgery and 33 open surgery. Mean age was 66.4 ± 4.1 years, 50% of patients were male. After a propensity score-matching, a similar total cost of EUR 18,297 ± 8,435 vs. 18,024 ± 7514 (p = 0.913) in robotic-assisted and open surgery groups was noted. Direct and indirect costs were similar in both groups. Robotic surgery showed higher operatory theatre-related costs (EUR 7532 ± 2,091 vs. 3351 ± 1872, p < 0.001), which were compensated by shorter hospital stay-related costs (EUR 4265 ± 4366 vs. 7373 ± 4698, p = 0.032). In the treatment of large ventral hernia, robotic surgery had higher operatory theatre-related costs, however, they were fully compensated by shorter hospital stays and resulting in similar total costs.

## Introduction

Ventral hernias are common and include different types of hernias, such as umbilical, paraumbilical, epigastric, Spigel, and incisional hernias^1^. If left untreated, these hernias can cause pain, discomfort, and potential complications. Incisional hernias develop after 10–15% of laparotomies, and the risk of recurrence increases with each repair attempt. Over the years, advances in surgical materials and techniques have improved the outcomes of ventral hernia repair procedures. Over 60% of ventral hernias are repaired using an open conventional approach, although there has been an exponential increase in repairs using minimally invasive techniques over the past decades^2^.

Laparoscopy and robotic surgery are less invasive techniques compared to open surgery. However, for large abdominal hernias, there might be additional challenges in laparoscopic or robotic repair. Factors like procedure complexity, tissue manipulation, the need for better access, and reduced visibility need to be taken into account. There are conflicting evidences and opinions within the scientific community regarding which method is better for treating large abdominal hernias. Some studies may suggest that robotic surgery could offer advantages in terms of precision and maneuverability compared to traditional laparoscopy; however, no clear clinical advantages in the treatment of simple hernias was demonstrated so far^3^. The lack of evidence demonstrating improved outcomes, coupled with the high costs in comparison to other techniques, renders the broader adoption of robotic platforms controversial^1,3^.

The choice of surgical technique to address large abdominal hernias depends on various factors such as the hernia's location, size, the patient's health status, and the surgeon's expertise. There are numerous research studies and discussions on surgical techniques for treating large abdominal hernias^2,4,5^. Studies reported higher costs for robotic than open surgery, mainly due to system acquisition and maintenance, equipment-instruments purchase, operative time and staff training^6,7^**.** To date, there are still no solid evidence-based references regarding the costs of treating large abdominal hernias, and the superiority of one technique over another remains uncertain.

The aim of our study was to compare the costs incurred by the hospital in patients treated for large ventral hernias operated either with robotic-assisted or open surgery.

## Methods

### Patient selection and data collection

Our institution is a specialized referral center for robotic-assisted abdominal wall surgery. From a prospectively maintained and audited database (Herniamed, www.herniamed.de), we searched for patients who underwent either robotic-assisted or open repair of large ventral hernias from January 2016 to December 2022. Patients were excluded if operated with a laparoscopic approach or in the setting of urgent surgery. Included patients were divided into two groups according to the surgical approach chosen.

The selection of the surgical approach was made by the attending surgeon after a comprehensive clinical assessment and a detailed assessment of preoperative CT scans, taking into account technical feasibility and surgical expertise. For both cases of open and robotic surgery, the operations were performed by the same experienced surgeons, meaning that they had more than 10 years of experience in general surgery and had conducted hundreds of abdominal wall operations using open, laparoscopic, and robotic surgery techniques. Patients within the robotic learning curve^8^ were not included in the analysis. The assessment of patients before surgery, the type of anesthesia chosen, surgical technique and mesh placement (i.e., retromuscular space), postoperative care, and cost procedures were the same for both groups. However, since 2018, we've noticed a shift from open surgery to robotic-assisted surgery. This means that most of the open surgeries were done in the initial years, while the majority of robotic-assisted surgeries have been done more recently. For included patients, we retrieved from the database demographics, clinical characteristics (i.e., age, sex, body mass index (BMI), presence of comorbidities and American Society of Anesthesia (ASA) score, surgical approach, type of hernia), postoperative complications according to the Clavien-Dindo classification^9^, length of hospital stay and costs.

The primary endpoint was to assess the costs incurred by the hospital in the robotic-assisted and open surgery groups.

### Definition of large ventral hernia

A ventral hernia was defined as a defect in the anterior fascia of the abdominal wall through which abdominal organs protrude. In the literature, several varying definitions of large ventral hernia have been proposed, yet a consensus was not found. The parameters commonly employed are the width, length, transverse dimensions, and the surface area calculation based on an ellipse. For the purpose of this study, we used the definition of large ventral hernia proposed by Katkhouda N, who defined a fascial defect measuring 7 cm or more in length^10^.

### Costs

We collected direct, indirect, and total cost data. Direct costs were those directly charged to patients such as medications, medical materials, physicians fees. Indirect costs were those calculated according to diagnostic and intervention codes for operating theatre, anesthesia, radiology, laboratory, salaries and hospital stay. Disposable and reusable robotic materials were directly charged to patients and were therefore calculated under medical materials (direct costs). A significant portion of robotic costs, such as installation, staff training, and maintenance, that could not be directly charged to patients, were distributed among patients who undergo robotic procedures and appeared as indirect costs under the operating theater expenses. Our policy dictates that the cost of purchasing the robotic system is distributed over a 10-year period and it is also accounted for as an indirect cost under the category Operating theater. Hospital costs were then reimbursed with the Swiss diagnosis-related group system^11^. Costs were expressed in euro (EUR). EUR / Swiss franc exchange rate: 0.957835 (updated on October 11th 2023).

Study approval was obtained by the local ethical committee (Comitato Etico Cantonale Ticino, registration number 2019–01132, Ref. CE 3495). Written informed consent and consent for publication were obtained from all patients. All methods were performed in accordance with the relevant guidelines and regulations. Strengthening the reporting of observational studies in epidemiology (STROBE) guidelines were followed^12^.

### Statistical analysis

Descriptive statistics were presented as absolute frequencies for categorical variables and mean with standard deviation (SD) for continuous variables. The comparison of categorical variables was carried out with the chi-square test, while for continuous variables the student t-test was used also to provide absolute difference with 95% confidence interval (95%CI). A propensity score–matched (PSM) analysis^13^ with 1:1 ratio was carried out according to age, sex, BMI, ASA score, type and dimension of the hernia. A p-value < 0.05 was considered statistically significant. MedCalc® Statistical Software version 22.014 was used (MedCalc Software Ltd, Ostend, Belgium,https://www.medcalc.org; 2023).

## Results

**Figure 1:**
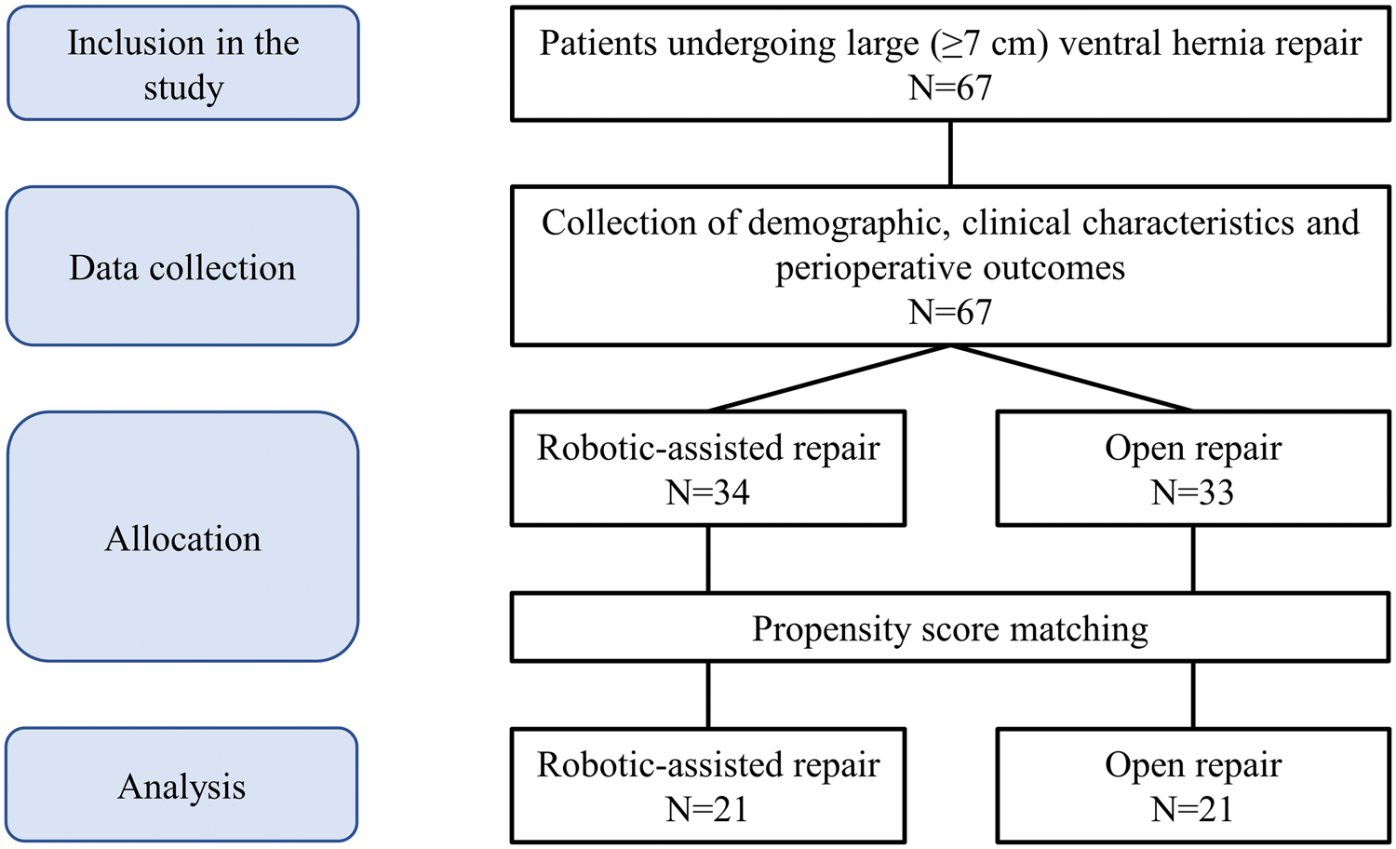
Study flow-chart

During the study period, a total of 67 patients were included in the analysis, with 34 in the robotic-assisted surgery group and 33 in the open surgery group. Figure 1. In all cases, the hernia was incisional, occurring at the site of a previous laparotomy. Significant differences between groups were noted in age, ASA score, and type of hernia, therefore, a propensity score-matching was performed to mitigate clinical disparities between the two groups. Among the initial 67 patients, 42 were successfully matched and included in the final analysis.

Demographic and clinical characteristics were found to be comparable in both groups. The mean age was 66.4 ± 4.1 years, and 11 patients (50%) were male. The mean body mass index (BMI) was 28.1 ± 1.0 kg/m^2^. Approximately 50% of the patients were classified as ASA ≥ III. Mean hernia defect length was 11.3 ± 2.9 cm, mean width was 4.7 ± 2.3 cm and mean ellipse area of the hernia defect was 43.6 ± 29.8 cm^2^. Mean mesh dimension was 718 ± 309 vs 636 ± 244 cm^2^ and all meshes were placed in the retromuscular space. Additional details for both unmatched and matched analyses can be found in Table 1.

**Table 1:** Patients’ characteristics.

	Overall analysis			Propensity score-matched analysis		
	Robotic surgery N = 34	Open surgery N = 33	p	Robotic surgery N = 21	Open surgery N = 21	p
Age, years (SD)	62.3 (12.9)	70.5 (12.0)	**0.009**	66.5 (10.8)	67.8 (13.0)	0.739
Sex, male (%)	15 (44.1)	18 (54.5)	0.397	10 (47.6)	12 (57.1)	0.541
Body mass index, kg/m^2^ (SD)	29.5 (5.9)	27.5 (5.0)	0.149	29.2 (4.7)	27.1 (5.3)	0.176
ASA score						
I, n (%)	2 (5.9)	0	**0.042**	0	0	0.759
II, n (%)	18 (52.9)	9 (27.3)		10 (47.6)	(42.9)	
III, n (%)	14 (41.2)	23 (69.7)		11 (52.4)	(57.1)	
IV, n (%)	0	1 (3.0)		0	0	
Comorbidities, n (%)						
Smoking, n (%)	9 (26.5)	6 (18.2)	0.419	4 (19.0)	4 (19.0)	1.000
Hypertension, n (%)	13 (38.2)	20 (60.6)	0.069	10 (47.6)	11 (52.4)	0.760
Diabetes mellitus, n (%)	8 (23.5)	13 (39.4)	0.165	6 (28.6)	6 (28.6)	1.000
Kidney disease, n (%)	1 (2.9)	5 (15.2)	0.082	0	3 (14.3)	0.076
Dyslipidemia, n (%)	3 (8.8)	3 (9.1)	0.970	2 (9.5)	2 (9.5)	1.000
Hernia type						
Median site, n (%)	27 (79.4)	32 (97.0)	**0.028**	21 (100)	20 (95.2)	0.317
Lateral site, n (%)	7 (20.6)	1 (3.0)		0	1 (4.8)	
Hernia dimension						
Length, cm (SD)	11.5 (3.4)	11.1 (2.3)	0.663	12.5 (3.7)	10.8 (2.2)	0.073
Width, cm (SD)	4.2 (2.0)	5.1 (2.6)	0.140	4.4 (2.2)	4.5 (2.0)	0.861
Area, cm^2^ (SD)	40.0 (25.8)	47.3 (33.4)	0.320	45.5 (29.4)	39.3 (22.6)	0.449
Mesh dimension						
Length, cm (SD)	30.1 (6.4)	28.8 (3.5)	0.322	32.0 (7.1)	28.7 (3.9)	0.074
Width, cm (SD)	22.9 (5.5)	21.6 (6.3)	0.370	24.5 (5.8)	21.6 (6.4)	0.137
Area, cm^2^ (SD)	718 (309)	636 (244)	0.236	816 (336)	635 (248)	0.054

ASA: American society of anesthesiology. Dichotomous variables are expressed as number with percentage. Continuous variables are expressed as mean with standard deviation (SD). Statistically significant values are reported in bold.

Postoperatively, 15 complications occurred, 5 in the robotic group and 10 in the open surgery group. In the robotic group, there was a case of Covid-19 infection (grade I Clavien-Dindo), two cases of postoperative ileus, a seroma conservatively treated (grade II Clavien-Dindo), and a case of hematoma that required reoperation (grade III Clavien-Dindo). In the open surgery group, there were three cases of mild respiratory distress, one case of pneumonia, and one case of ileus, all treated with medications (grade II Clavien-Dindo). Additionally, three cases of seroma requiring radiological drainage and one case of evisceration requiring reoperation were observed (grade III Clavien-Dindo), along with one case of acute lower limb ischemia that required an operation (grade IV Clavien-Dindo). The length of hospital stay was 8.9 ± 10.5 days. Table 2.

**Table 2:** Postoperative results.

	Overall analysis			Propensity score-matched analysis		
	Robotic surgery N = 34	Open surgery N = 33	p	Robotic surgery N = 21	Open surgery N = 21	p
Postoperative complications						
Grade I, n (%)	1 (2.9)	0	0.289	0	0	0.459
Grade II, n (%)	3 (8.8)	5 (15.2)		2 (9.5)	5(23.8)	
Grade III, n (%)	1 (2.9)	4 (12.1)		1 (4.8)	1(4.8)	
Grade IV, n (%)	0	1 (3.0)		0	0	
Length of hospital stay, days (SD)	5.1 (4.0)	13.0 (13.3)	**0.002**	5.3 (4.5)	10.4 (5.2)	**0.002**

Dichotomous variables are expressed as number with percentage. Continuous variables are expressed as mean with standard deviation (SD). Postoperative complications were graded according to the Clavien-Dindo classification. Statistically significant values are reported in bold.

The descriptive cost analysis was carried out for patients included in the PSM analysis. The results revealed a similar total cost in both robotic and open surgery groups of EUR 18,297 ± 8435 vs. 18,024 ± 7514 (absolute difference EUR − 272, 95%CI − 5254–4710, p = 0.913). Overall, direct costs were EUR 1469 ± 1302 and indirect costs were EUR 16,599 ± 7337. Between the groups, no significant difference regarding direct costs was noted and they were EUR 1402 ± 1219 vs. 1536 ± 1407 (absolute difference EUR + 134, 95%CI − 687–954, p = 0.744). Indirect costs were EUR 16,895 ± 7926 vs. 16,304 ± 6881 (absolute difference EUR − 590, 95%CI − 5220–4039, p = 0.798). Among indirect costs, operating theater (EUR 7532 ± 2091 vs. 3351 ± 1872, absolute difference EUR − 4180, 95%CI − 5418 to − 2942, p < 0.001) and anesthesia costs (EUR 2906 ± 684 vs. 1770 ± 890, absolute difference EUR − 1136, 95%CI − 1631 to − 641, p < 0.001) were significantly higher in patients undergoing open surgery. On the other hand, due to the shorter hospital stay in the robotic surgery group, the hospitalization (EUR 4265 ± 4366 vs. 7373 ± 4698, absolute difference EUR + 3108, 95%CI 280–5937, p = 0.032) and medical salary costs (EUR 613 ± 650 vs. 1547 ± 1213, absolute difference EUR 933, 95%CI 326–1540, p = 0.003) were significantly lower in the robotic surgery group. In summary, in the robotic surgery group the higher operation-related costs were fully compensated by lower hospitalization-related costs and both indirect costs and total costs were similar between groups. Further details are presented in Table 3.

**Table 3:** Cost analysis.

	Cost analysis		
	Robotic surgery N = 21	Open surgery N = 21	p
Total costs, EUR (SD)	18,297 (8435)	18,024 (7514)	0.913
Direct costs, EUR (SD)	1402 (1219)	1536 (1407)	0.744
Medications, EUR (SD)	167 (199)	343 (552)	0.178
Medical materials, EUR (SD)	1966 (1539)	1659 (1931)	0.572
Medical salaries, EUR (SD)	252 (729)	352 (756)	0.663
Indirect costs, EUR (SD)	16,895 (7926)	16,304 (6881)	0.798
Operating theater, EUR (SD)	7532 (2091)	3351 (1872)	** < 0.001**
Administration, EUR (SD)	294 (165)	248 (92)	0.267
Intensive care unit, EUR (SD)	746 (2007)	1011 (2917)	0.734
Anesthesia, EUR (SD)	2906 (684)	1770 (890)	** < 0.001**
Radiology and laboratory, EUR (SD)	402 (663)	997 (1525)	0.109
Hospitalization costs, EUR (SD)	4265 (4366)	7373 (4698)	**0.032**
Medical salaries, EUR (SD)	613 (650)	1547 (1213)	**0.003**
Physiotherapy, EUR (SD)	137 (206)	192 (198)	0.376

Continuous variables are expressed as mean with standard deviation (SD). Costs were expressed in euro (EUR). EUR/Swiss franc exchange rate: 0.957835 (updated on October 11th 2023). Statistically significant values are reported in bold.

## Discussion

In the present study regarding the cost-effectiveness of different techniques in large ventral hernia repair, we compared patients undergoing robotic-assisted approach with those treated with open surgery. According to our results, the total cost of patient hospitalization after robotic-assisted surgery is similar to the cost of hospitalization after open repair. In fact, the higher surgical procedure and anesthesia costs of robotic surgery were fully compensated by shorter hospital stay and lower medical salary fees.

For decades the only available surgical approach to treat large ventral hernia was open surgery^5^. Laparoscopic surgery was subsequently proposed and demonstrated positive results. Indeed, the laparoscopic treatment of ventral hernia has been described as a safe and feasible technique with a low recurrence rate, applicable to nearly all patient populations, including those with obesity and multiple previous surgeries^14^. Several advantages of laparoscopy have been described so far in the literature,however, this approach has been reported to be not completely free of difficulties and complex problems, some of which are hard to solve^15,16^. The introduction of robotic surgery into clinical practice has overcome some challenges of the laparoscopic approach. In this regard, the most encouraging aspect of robots is the chance of combining the concepts of open repair (component separation, fascial closure, mesh fixation and, when needed, transversus abdominis release) with the benefits of minimally-invasive surgery. In fact, the robotic system enhances dexterity by employing articulated instruments with a broader range of motion, thereby increasing flexibility and capacity to adapt to diverse anatomical challenges. This makes robotic surgery well-suited for complex procedures where traditional methods may encounter limitations^17,18^. In our retrospective series, almost no patients treated laparoscopically for large ventral hernia were retrieved. Indeed, over the past 7 years, our center has transitioned from open to robotic surgery. Our development follows a trend well established in robotic centers worldwide, supported by the literature indicating the safety and feasibility of the robotic approach for complex ventral hernia surgeries. Bittner et al.^19^ found that robotic-assisted transversus abdominis release for complex ventral hernia repair offered low morbidity and decreased hospital length of stay compared to open transversus abdominis release. Gonzalez A et al.^20^ described that the robotic-assisted ventral hernia repair has reproducible safety and performance, with similar short-term outcomes compared to laparoscopic and open ventral hernia repair. Similar findings were also recently reported by Bauer K et al.^21^ who reported that robotic-assisted hernia repair is safe and practical for complex hernias. In our experience, the safety and feasibility of the robotic approach was supported by the absence of intraoperative conversion to open surgery and similar postoperative outcomes compared to open surgery. Moreover, in our series we reported a shorter hospital stay in patients undergoing robotic surgery.

However, potential drawbacks of robotic surgery could include issues related to its availability, the varied experience levels among surgeons and costs. Ye L et al.^4^ reported that robotic ventral hernia repair is more expensive than laparoscopic repair, but not significantly different from open surgery in terms of cost. Indeed, the literature seem to support the laparoscopy particularly for non-complex hernias as it may be more cost-effective than robotic^22^. However, we decided to include in our series only patients with complex ventral hernias, adopting the definition of Katkhouda N^10^. In fact, applying laparoscopy to these patients might be challenging or not possible at all. For these complex situations robotic surgery offers many advantages and should be compared to open surgery. Nguyen B et al.^23^ published an article reporting that robotic-assisted repair of complex ventral hernia repairs has shorter hospital stays and better patient outcomes compared to open repair. Also, Greenberg J et al.^24^ reported that robotic retromuscular ventral hernia repair was associated with shorted length of stay and decreased surgical site infections compared to an open approach. Clinical advantages of robotic surgery led to a compensation of robotic-related costs. Dauser B et al.^25^ reported that the robotic-assisted approach for complex ventral hernia repair is a viable option with higher procedure-related costs offset by a significant shorter hospital stay. Also, Warren JA et al.^26^ found that robotic retromuscular ventral hernia repair has similar hospital cost (United States dollars 13,943 vs. 19,532) compared to open repair. On the other hand, it should be considered that costs of robotic surgery are expected to decrease in the future, Awad et al.^27^ reported that increasing surgeon experience with robotic hernia repair is associated with a predictable reduction in operative time, complication rates, and direct operative cost per case.

In our experience, the cost analysis yielded noteworthy results. Our results demonstrate the validity of both surgical techniques, with a total cost close to EUR 18,000 for each approach. Operating room costs remain significantly higher, with an average cost of approximately EUR 7532 for robotic-assisted surgery compared to EUR 3351 for open surgery. However, it is crucial to highlight substantial differences in indirect cost analysis. Notably, robotic surgery has reduced the costs and intensive care unit stays by about 50%, with an expenditure of approximately EUR 746 per stay for robotic-assisted surgery compared to EUR 1011 per stay for open surgery. With fewer postoperative complications, there is a reduced need for additional radiological examinations and laboratory analyses, further contributing to cost reduction. This leads to a significant decrease in total hospitalization costs, averaging around EUR 4265 for robotic surgery compared to EUR 7373 for open surgery. The significantly higher costs for operating theatre and anesthesia in the robotic group was fully compensated by lower hospitalization-, complication-related and medical salary costs. The rather long hospital stay was justified in our series by the fact that most patients were aged over 65 years, overweight, and had multiple comorbidities. All hernias were large and required extensive abdominal wall dissections. Moreover, the rate of postoperative complications was not negligible, as 15 out of 67 (22.4%) complications occurred. We believe our series reflects the clinical experience of many centers performing challenging hernia operations, and as a PSM analysis was carried out, it is reasonable to assume that these factors equally impacted the costs of both groups. Therefore, total costs were similar for robotic and open surgery, but with the clinical advantage of a shorter hospital stay in the robotic group.

From a technical perspective, it is essential to note that the costs and durations of robotic surgery remain dependent on the skill level of the surgical team. In our analysis, we utilized surgeries performed by expert robotic surgical teams (4). This emphasizes the fundamental role of surgical expertise, as achieving competence in robotic abdominal wall surgery takes time, and a realistic learning curve should be considered. This curve can vary depending on the individual surgeon's experience and training program. In our series, we included only operations performed by the same experienced surgeons to minimize the bias related to the learning curve. These surgeons, who had operated on ventral hernias in an open fashion for years, switched to robotic techniques after undergoing structured training and gained experience over time. The first patient in the robotic group underwent surgery 2 years after the implementation of the robotic system in our center. Moreover, our robotic operating room is equipped with the Da Vinci Xi dual console, facilitating training in a quiet and efficient environment while gradually exposing trainees with varying levels of competence to robotic surgery. Adequately trained nursing staff is equally critical, and at our institution, with over 450 robot-assisted procedures annually, nearly all operative nurses are familiar and comfortable with this technology.

It is important to acknowledge limitations of this study. First, it is a single-center, retrospective design, introducing inherent measurement bias. Second, a precise definition of large ventral hernias remains lacks, making it challenging to consider a broader patient population for the study. The analysis included different surgeons performing robotic and open surgery, no operation was carried out within the learning curve, but different surgical skills may have affected operative times and, therefore, costs. Costs of robotic were attributed to patients as direct or indirect costs and included medical materials, installation, staff training, and maintenance. The cost of purchase was also included in the analysis, as the initial investment was distributed over a 10-year period and charged to robotic patients as an indirect cost. According to the volume of operations carried out through the years, we may roughly estimate that these costs range between EUR 500–800 per patient. Also, patients included in the two groups were not similar as it is a retrospective analysis. However, we accounted for their differences by employing a thorough propensity score matching analysis. The relatively small sample size limited further subgroup analyses or a more comprehensive propensity score matching. The sample size constrained solid conclusions from a clinical point of view, but should reliably depict economical aspects.

We believe our study offers a reliable description of costs incurred by hospitals for treating patients with large ventral hernia with either a robotic or an open approach. Our results are generalizable to surgical departments carrying out complex abdominal wall surgeries with robotic platforms. Care must be taken in applying our results to simple hernias, where standard laparoscopy might be more cost-effective than robotics.

## Conclusions

Despite limitations, our study showed that in the treatment of large ventral hernia, robotic surgery had higher operatory theatre-related costs, however, they were fully compensated by shorter hospital stays and resulting in similar total costs. Further studies addressing cost-effectiveness and overall social costs of robotic-assisted surgery for large ventral hernias and are needed to confirm our preliminary findings.

## Data Availability

The dataset analysed during the current study is available from the corresponding author on request.
